# The nucleocapsid protein facilitates p53 ubiquitination-dependent proteasomal degradation *via* recruiting host ubiquitin ligase COP1 in PEDV infection

**DOI:** 10.1016/j.jbc.2024.107135

**Published:** 2024-03-04

**Authors:** Wanyu Dong, Yahao Cheng, Yingshan Zhou, Jingmiao Zhang, Xinya Yu, Haicun Guan, Jing Du, Xingdong Zhou, Yang Yang, Weihuan Fang, Xiaodu Wang, Houhui Song

**Affiliations:** Key Laboratory of Applied Technology on Green-Eco-Healthy Animal Husbandry of Zhejiang Province, Zhejiang Provincial Engineering Laboratory for Animal Health Inspection & Internet Technology, Zhejiang International Science and Technology Cooperation Base for Veterinary Medicine and Health Management, China-Australia Joint Laboratory for Animal Health Big Data Analytics, College of Animal Science and Technology & College of Veterinary Medicine of Zhejiang A&F University, Hangzhou, China

**Keywords:** porcine epidemic diarrhea virus (PEDV), N protein, ubiquitin ligase COP1, interaction, p53 degradation

## Abstract

Porcine epidemic diarrhea virus (PEDV) is a highly contagious enteric pathogen of the coronavirus family and caused severe economic losses to the global swine industry. Previous studies have established that p53 is a host restriction factor for PEDV infection, and p53 degradation occurs in PEDV-infected cells. However, the underlying molecular mechanisms through which PEDV viral proteins regulate p53 degradation remain unclear. In this study, we found that PEDV infection or expression of the nucleocapsid protein downregulates p53 through a post-translational mechanism: increasing the ubiquitination of p53 and preventing its nuclear translocation. We also show that the PEDV N protein functions by recruiting the E3 ubiquitin ligase COP1 and suppressing COP1 self-ubiquitination and protein degradation, thereby augmenting COP1-mediated degradation of p53. Additionally, COP1 knockdown compromises N-mediated p53 degradation. Functional mapping using truncation analysis showed that the N-terminal domains of N protein were responsible for interacting with COP1 and critical for COP1 stability and p53 degradation. The results presented here suggest the COP1-dependent mechanism for PEDV N protein to abolish p53 activity. This study significantly increases our understanding of PEDV in antagonizing the host antiviral factor p53 and will help initiate novel antiviral strategies against PEDV.

Porcine epidemic diarrhea (PED), caused by porcine epidemic diarrhea virus (PEDV), is a highly contagious intestinal disease characterized by vomiting, acute watery diarrhea, dehydration, and high mortality rate in newborn piglets (1). PED was initially identified in England in 1978 (2) and subsequently discovered across most of Europe (3). In 2010, a highly pathogenic PEDV variant strain emerged in China and caused a large scale of PED outbreaks (4, 5, 6). Later, this strain spread rapidly to numerous countries, resulting in substantial economic losses to the global swine industry (7, 8).

PEDV is an enveloped virus with single-stranded positive-sense RNA genome of approximately 28,000 nt that belongs to the *Alphacoronavirus* genus within the Coronaviridae family. Its genome is arranged in the following order: 5′-ORF1a-ORF1b-S-ORF3-E-M-N-3′, encoding 16 nonstructural proteins (nsp1–nsp16), four structural proteins (spike [S], envelope [E], membrane [M], nucleocapsid [N]), and an accessory protein ORF3 (7). The N protein is the major structural component of the virion and has multiple functions in viral infection (9, 10, 11, 12, 13). Its primary role is to organize the viral genome and enhance the efficiency of virus transcription and assembly (14). In addition to its function in genome management, PEDV N protein appears to modulate some cellular processes related to viral survival (15), protect host cells from induced apoptosis (16, 17), and antagonize type I interferon (IFN) production by direct interaction with TBK1 and suppression of IFN regulatory factor 3 (18). PEDV N protein has cell cycle-regulatory functions by direct interaction with p53 (19). These studies suggest that N protein is involved in multiple layers of complex functional roles during PEDV infection.

The tumor-suppressor protein p53 regulates a plethora of target genes by triggering multiple biological processes such as apoptosis, cell-cycle arrest, and DNA repair (20, 21). p53 is also identified as a direct target gene of the type I IFN (IFNα/β) pathway, and thus, its role in antiviral innate immunity is equally established (22). Proteasomal degradation of p53 is regulated by several E3 ubiquitin ligases such as MDM2, Pirh2, or COP1 (23). Ablation of COP1 or MDM2 increased the half-life of p53 at comparable levels, both being more effective than Pirh2 ablation (24). p53-dependent apoptosis and IFN response have been considered as a powerful control to limit viral infection, for example, restricting the infections by vesicular stomatitis virus (25), influenza A virus (26), and PEDV (27). However, many viral N proteins have been shown to suppress p53 functions by using the cellular ubiquitination machinery. The spring viraemia of carp virus uses its N protein to bind and degrade host p53 through suppressing the K63-linked ubiquitination (28). Hantaan virus N protein initiates MDM2-dependent degradation of p53 (29). Influenza A virus N protein targets RNF43 to modulate p53 ubiquitination levels (30).

p53 is known as a key player in inducing strong antiviral defenses against PEDV (27), while PEDV infection decreased p53 expression in HEK293T cells and in Vero cells (27, 31). These findings suggest that PEDV could antagonize the p53-induced antiviral responses. The molecular mechanisms by which PEDV viral proteins regulate p53 degradation remains unclear. In this study, we provide evidence that PEDV N protein is capable of downregulating p53 levels through ubiquitin-proteasome pathway by recruiting E3 ubiquitin ligase COP1 and preventing the nuclear translocation of p53. The N-terminal domain (NTD) of N protein, which is pivotal for COP1 binding, is demonstrated to be required for p53 degradation. These findings provide insights into novel functions of PEDV N protein and potential new targets for the development of antiviral drugs against PEDV or other coronaviruses.

## Results

### p53 was degraded through the proteasome pathway in the late stage of PEDV infection

**Figure 1:**
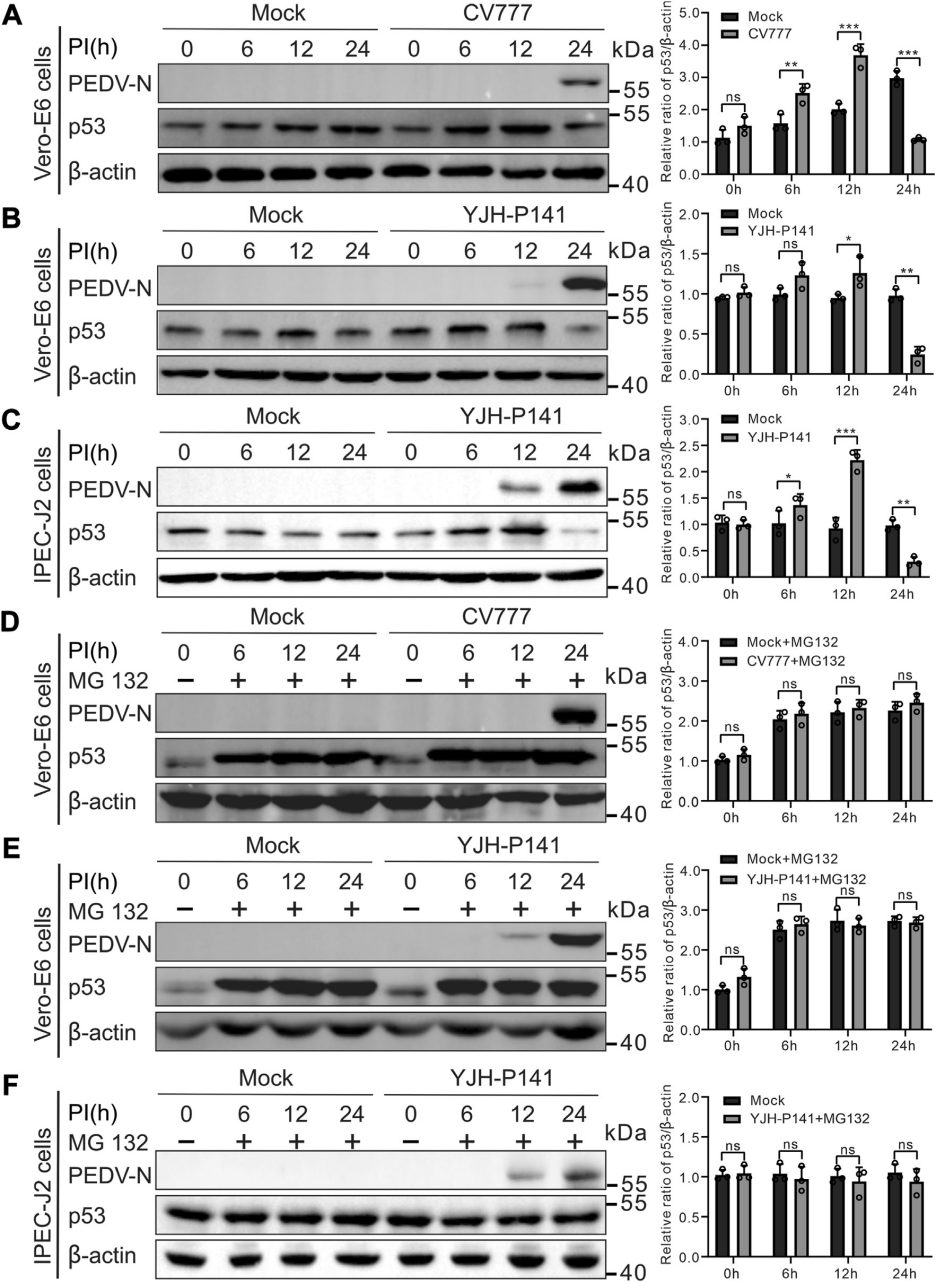
**PEDV infection inhibits p53 expression through the proteasome pathway.***A*–*C*, the p53 expression was downregulated upon PEDV infection at 24 h postinfection (hpi). The Vero E6 cells were infected with PEDV CV777 or YJH-P141 at  0.01 MOI and harvested at indicated time points (*A* and *B*). The IPEC-J2 cells were infected with PEDV YJH-P141 at  0.1 MOI and harvested at indicated time points (*C*). Endogenous p53 expression was determined by Western blotting. *D*–*F*, PEDV-induced p53 degradation was blocked by MG132 treatment. Vero E6 cells were infected with 0.01 MOI of PEDV CV777 or YJH-P141 (*D* and *E*). IPEC-J2 cells were infected with 0.1 MOI of PEDV YJH-P141 (*F*). Cells were collected at the indicated times and treated with the proteasome inhibitor MG132 (10 μM) for 4 h. p53 and PEDV N protein levels were assessed *via* Western blotting. Bands were quantitated by densitometry using ImageJ software, and p53 expression was standardized to that of β-actin. The results in the bar charts on the right represent mean ± SD of densitometric data from three independent experiments. The *p*-values were estimated with Student’s *t*-tests (∗*p* < 0.05, ∗∗*p* < 0.01, ∗∗∗*p* < 0.001). PEDV, porcine epidemic diarrhea virus.

To investigate the effect of PEDV infection on p53 expression, Vero E6 cells were infected with a classical strain CV777 for evaluation of total p53 protein by Western blotting at 0, 6, 12, and 24 h postinfection (hpi). As the results showed that PEDV infection upregulated p53 expression at 6 and 12 hpi but downregulated markedly at 24 hpi as compared with the mock-infected cells (Fig. 1*A*). We next examined whether PEDV-induced p53 downregulation was limited to the specific virus strain used. To this end, we repeated these experiments with PEDV YJH-P141 strain. We found that a field PEDV strain YJH-P141 also showed a similar pattern of p53 changes over time, significantly decreased at 24 hpi (Fig. 1*B*). To further confirm this observation, IPEC-J2 cells were infected with PEDV YJH141 at the indicated time points. Similar results were observed in IPEC-J2 cells (Fig. 1*C*). These results demonstrate that PEDV interferes with expression or stability of the host p53 protein.

The proteasome pathway has been recognized as the major pathways involved in intracellular p53 degradation (32). To determine if PEDV induced downregulation of p53 is proteasome-dependent, both Vero E6 and IPEC-J2 cells were subjected to infection with PEDV as well as treated with proteasome inhibitor MG132. Western blotting analysis suggests that MG132 suppressed the PEDV-induced p53 reduction (Fig. 1, *D*–*F*), indicating that PEDV infection could promote proteasome degradation of p53.

### PEDV N protein promoted p53 degradation *via* the ubiquitin-proteasome pathway

**Figure 2:**
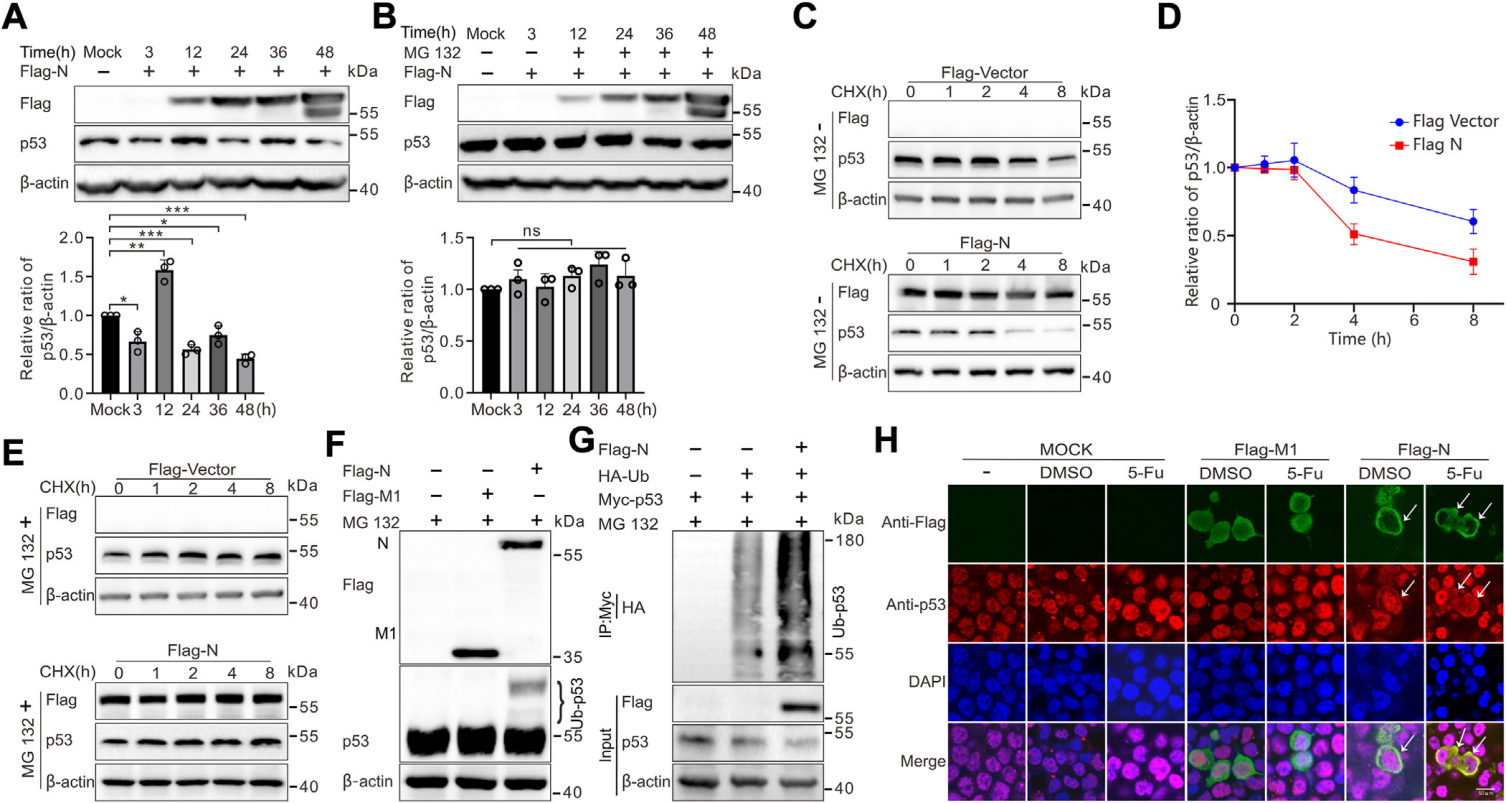
**PEDV N protein destabilizes the p53 and enhances p53 ubiquitination.***A*, PEDV N protein decreased the steady-state level of p53. HEK293T cells were transfected with plasmids expressing flag-tagged N protein for indicated hours, the levels of p53 were determined by Western blotting. *B*, PEDV N protein-induced p53 declination was rescued by the proteasome inhibitor MG132. HEK293T cells overexpressing Flag-tagged N protein were further cultured in the presence or absence of MG132 for various time points and the whole cell lysates were collected for Western blotting with corresponding antibodies. *C*–*E*, the PEDV N protein accelerated p53 turnover. HEK293T cells were transfected with expression plasmids for flag-tagged N or with the flag-tagged vector. At 24 h posttransfection (hpt), cells were treated with either 100 μg/ml CHX alone or CHX with MG132 for the indicated periods. Western blotting was then performed using the designated antibodies on the total lysates. *F*, PEDV N protein increased ubiquitination of endogenous p53. HEK293T cells were seeded in 10-cm^2^ dishes and transfected with 2 μg Flag-N or 2 μg Flag-M1. At 20 hpt, the cells were treated with 10 μM of MG132 for 4 h and then analyzed by Western blotting with corresponding antibodies. *G*, PEDV N protein promoted ubiquitination of exogenous p53. HEK293T cells were cotransfected with 2 μg Flag-N, 2 μg Myc-p53, and 2 μg HA-Ub and treated with MG132 (10 μM) for 4 h before harvesting at 24 hpt. p53 was immunoprecipitated with anti-Myc and immunoblotted with anti-HA. *H*, PEDV N protein trapped p53 in the cytoplasm. HEK293T cells were transfected with 2 μg Flag-N or 2 μg Flag-M1 control plasmid. After 20 hpt, cells were treated with DMSO (10 μM) or 5-Fu (10 μM) for 4 h, fixed with 4% paraformaldehyde and stained with DAPI for nuclei. The anti-goat secondary antibody conjugated to Alexa-555 for p53 (*red*) and Alexa-488 for Flag (*green*) were used. The cells were observed under confocal microscope. The scale bar is 50 μm. *White arrows* indicate perinuclear colocalization (*yellow* fluorescence) of the two proteins in a subset of HEK293T cells in the merged panel. Densitometric analysis of p53 relative to β-actin using ImageJ. Data are expressed as mean ± SD of triplicate samples. *p*-values were estimated with Student’s *t*-tests (∗*p* < 0.05, ∗∗*p* < 0.01, ∗∗∗*p* < 0.001). “ns” means no significant difference. CHX, cycloheximide; Flag-N, flag-tagged N protein; PEDV, porcine epidemic diarrhea virus.

The shuttling between the nucleus and cytoplasm of coronavirus N protein provides clues to ascertain the importance of N protein in p53 stabilization (15). To determine whether PEDV N plays an important role in regulating p53 degradation, plasmids expressing flag-tagged N protein (Flag-N) were transfected into HEK293T cells for different hours. Figure 2*A* shows that p53 protein increased at 12 h posttransfection (hpt) but was lower at 24 to 48 hpt than that at 12 hpt. Treatment of proteasome inhibitor MG132 reduced p53 degradation in PEDV N protein-expressing cells (Fig. 2*B*), proving that PEDV N protein utilized proteasomal pathway to promote p53 degradation.

To investigate the effect of N protein on the rate of p53 protein degradation, HEK293T cells expressing N protein were subjected to treatment with the protein synthesis inhibitor cycloheximide (CHX) in the presence or absence of MG132 for different time periods at 24 hpt. We observed more significant reduction of p53 in N protein-expressing and CHX-treated cells, as compared with CHX-treated cells without N protein expression (Fig. 2, *C* and *D*). Furthermore, p53 degradation was abolished in the N protein-expressing cells in the presence of MG132 (Fig. 2*E*). The results showed that the stability of the p53 protein was significantly reduced by PEDV N protein.

In order to further explore whether N protein-mediated proteasome degradation of p53 is the consequence of ubiquitination, we overexpressed Flag-N or Flag-M1 (as control) in HEK293T cells for *in vivo* ubiquitination assays. Figure 2*F* reveals that increased p53 ubiquitination only upon Flag-N expression, suggesting that PEDV N increases the endogenous p53 ubiquitination. Then, we tested the effect of N protein on p53 ubiquitination by co-expression of HA-tagged ubiquitin (HA-Ub), Myc-tagged p53 (Myc-p53), and Flag-N in HEK293T cells. The ubiquitin-based immunoprecipitation assay showed that p53 ubiquitination was strikingly enhanced in the cells that overexpress N protein as compared with the control cells (Fig. 2*G*). In addition, to visualize the effect of N protein on p53 ubiquitination, a similar immunofluorescence staining strategy was deployed. In the cells expressing Flag-N treated either with 5-fluorouracil (5-Fu) or with reagent control dimethylsulfoxide (DMSO), there was apparent accumulation of p53 around the nuclear membrane. Perinuclear accumulation of p53 was even more pronounced when the N-expressing cells was stimulated with 5-Fu (white arrows, Fig. 2*H*), a known activator of p53 nuclear entry (33). However, in the cells expressing Flag-M1 (for comparative purpose as a “negative” protein), there was no apparent p53 accumulation around the nuclear membrane, even upon 5-Fu treatment. These findings suggest that PEDV N acts to trap p53 in the cytoplasmic compartment. Altogether, these results clearly reveal that PEDV N promotes p53 degradation *via* the ubiquitin-proteasome pathway and directly leads to cytoplasmic accumulation.

### COP1 is the E3 ubiquitin ligase involved in N-mediated p53 degradation

**Figure 3:**
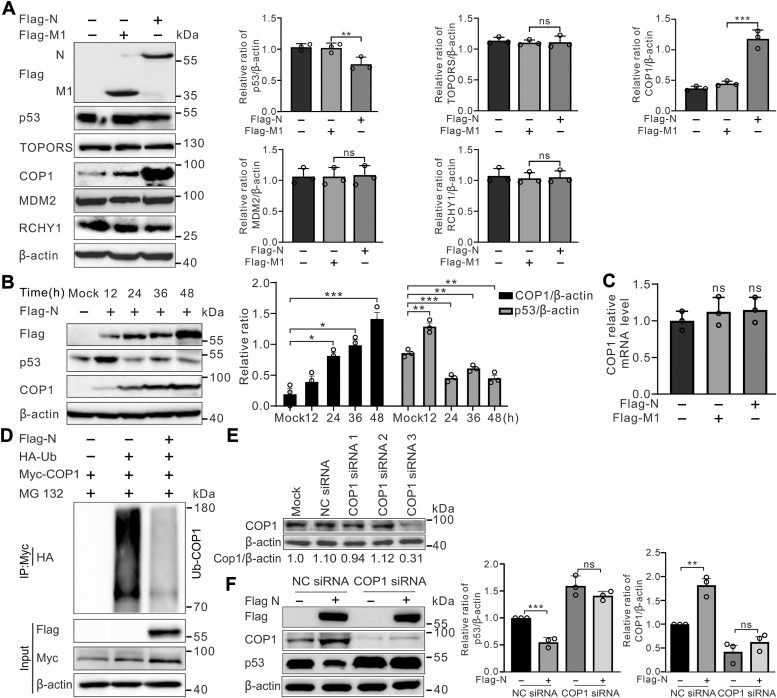
**COP1 is the E3 ubiquitin ligase involved in the degradation of p53 induced by PEDV N protein.***A*, PEDV N upregulated COP1 protein levels. Plasmids Flag-N or Flag-M1 (2 μg each) was transfected into HEK293T cells. After 24 h, total lysates were immunoblotted to detect the expression of p53, TOPORS, COP1, MDM2, and RCHY1. Densitometric analysis of p53, TOPORS, COP1, MDM2, and RCHY1 relative to β-actin using ImageJ in the right panel. *B*, PEDV N expression increased the steady-state level of COP1. HEK293T cells were transfected with Flag-tagged N plasmids for indicated hours, the levels of p53 and COP1 were determined by Western blotting. Densitometric analysis of COP1 or p53 relative to β-actin using ImageJ in the *right panel*. *C*, Flag-N expression had no effects on *cop1* mRNA levels. HEK293T cells transfected with Flag-M1 or Flag-N plasmids were subjected to RNA extraction, reverse transcription, and real time quantitative PCR (RT-qPCR) to analyze *cop1* gene expression. Results were normalized for GAPDH expression. *D*, PEDV N protein decreased ubiquitination of COP1. HEK293T cells were cotransfected with Flag-N, Myc-COP1, and HA-Ub and treated with MG132 (10 μM) for 4 h before harvesting at 24 hpt. COP1 was immunoprecipitated with anti-Myc and immunoblotted with anti-HA. *E*, the efficiency of the COP1 siRNA was assessed by Western blotting. HEK293T cells were transfected with the indicated siRNA. At 24 h posttransfection, cell lysates were detected with anti-COP1 or anti-β-actin antibody. COP1 levels were quantified and normalized to the amount of β-actin. *F*, N-induced p53 degradation was suppressed in COP1 knockdown cells. HEK293T cells were transfected with either negative siRNA or COP1 targeting-specific siRNAs for 24 h, followed by subsequent transfection with Flag-tagged N protein. At 24-h posttransfection, cells were harvested and detected using Western blotting with the indicated antibodies. Densitometric analysis of COP1 or p53 relative to β-actin using ImageJ in the *right panel*. Data are expressed as mean ± SD of triplicate samples (∗*p* < 0.05, ∗∗*p* < 0.01, ∗∗∗*p* < 0.001). “ns” means no significant difference. Flag-N, flag-tagged N protein; PEDV, porcine epidemic diarrhea virus.

There are an increasing number of E3 ligases implicated in regulation of p53 ubiquitination, including MDM2, RCHY1, COP1, and TOPORS (34, 35) which directly interact with p53 and target p53 for proteasome-mediated degradation in a ubiquitin-dependent fashion. As shown previously, we confirmed that PEDV N could induce p53 degradation in a ubiquitin–proteasome–dependent manner. However, being a nucleocapsid protein of PEDV, N protein itself lacks any of the E3 ubiquitin ligase domains. To explore which E3 ubiquitin ligase was involved, we measured the E3 ubiquitin ligases in Flag-N-expressing HEK293T cells by Western blotting. As a result, only COP1 was markedly increased as compared to the control cells. Conversely, no apparent differences were observed in the expression of MDM2, TOPORS, and RCHY1 (Fig. 3*A*). Furthermore, N protein increased the expression of COP1 in a time-dependent manner (Fig. 3*B*). Most interestingly, inconsistent with the COP1 protein expression level, its mRNA level was steady in the cells transfected with Flag-N, in comparison with the levels in Flag-M1 transfected and untransfected cells evaluated by RT-qPCR (Fig. 3*C*), suggesting that the effect of N protein on COP1 was not due to changes in transcription but to stabilization of COP1. In addition, the effect of N protein on COP1 ubiquitination was examined. As shown in Figure 3*D*, COP1 polyubiquitination was reduced in N-overexpressing cells compared to control cells, suggesting that N protein increases COP1 stability by inhibiting COP1 ubiquitination.

To further implicate COP1 in N-mediated p53 degradation, we synthesized three distinct pairs of small interfering RNA (siRNA) targeting the *cop1* gene and evaluated its effect on p53 degradation. Among the three synthesized siRNAs, siCOP1-3 was the most potent in downregulating endogenous COP1 levels (Fig. 3*E*). Consequently, siCOP1-3 was selected for the subsequent knockdown experiments. As demonstrated in Figure 3*F*, depletion of COP1 significantly suppressed N-induced p53 degradation. These findings suggest that the E3 ubiquitin ligase COP1 is involved in N-mediated degradation of p53.

### PEDV N protein interacted with COP1

**Figure 4:**
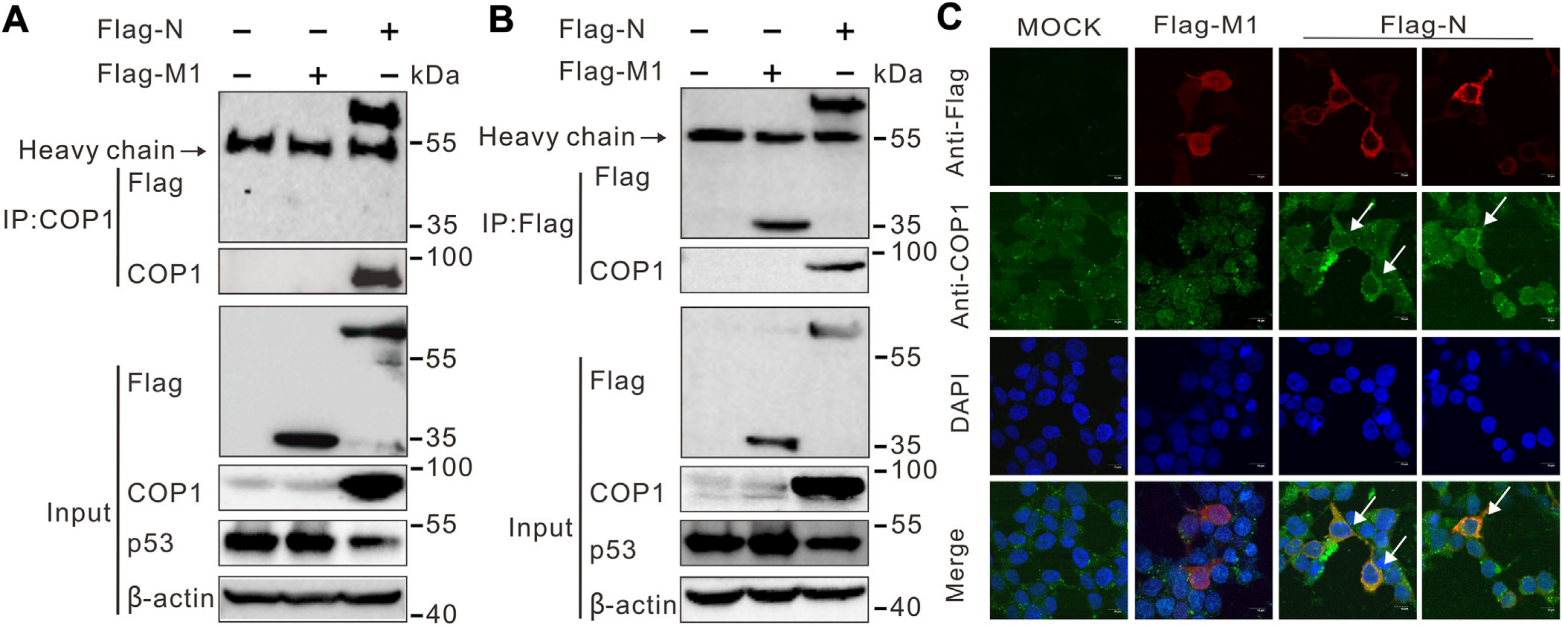
**PEDV N protein interacts with COP1.***A* and *B*, N protein coprecipitated with endogenous COP1. Plasmids that encoded Flag-N or Flag-M1 were transfected into HEK293T cells and harvested at 24 h. 10% of the lysed cells were saved as input, and the remaining portions were subjected to immunoprecipitation with 1 μg anti-Flag antibody (*A*) or 1 μg anti-COP1 antibody (*B*). The presence of COP1 proteins or N proteins in the immunoprecipitates was assessed by Western blotting against anti-COP1 or Flag antibodies. *C*, N protein colocalized with COP1 in cytoplasm. HEK293T cells were transfected with Flag-tagged N or Flag-tagged N for 24 h and fixed 4% paraformaldehyde. Ectopically expressed N and endogenous COP1 were detected using Flag antibody (1:1000 dilution) and COP1 (1:500 dilution), respectively, followed by anti-mouse Alexa Fluor 555 (*red*) and anti-mouse Alexa Fluor 488 (*green*). Cell nuclei were stained with DAPI. The images were sequentially captured using an Olympus confocal microscope. The scale bar is 10 μm. *White arrows* indicate perinuclear colocalization (*yellow* fluorescence) of the two proteins in a subset of HEK293T cells in the merge panel. Flag-N, flag-tagged N protein; PEDV, porcine epidemic diarrhea virus.

Viral proteins are found to promote ubiquitination and proteasomal degradation of p53 tumor suppressor protein by binding with E3 ligase (36). To investigate if COP1 is also involved in N protein–mediated p53 ubiquitination and degradation by binding with PEDV N, lysates from HEK293T cells overexpressing Flag-tagged N or Flag-tagged M1 as a control vector were subjected to immunoprecipitation with anti-Flag and immunoblotted with anti-COP1 or vice versa. The results showed that Flag-N and endogenous COP1 were able to bring down each other (Fig. 4, *A* and *B*, lane 3), which demonstrated the interactions between PEDV N protein and COP1. Flag-tagged M1 was used as negative control (Fig. 4, *A* and *B*, lane 2). To further confirm the interaction between PEDV N and COP1, the subcellular distributions of PEDV N and COP1 proteins were observed under confocal microscopy. The immunofluorescence images revealed that COP1 protein was colocalized with PEDV N protein in the cytoplasm of HEK293T cells (Fig. 4*C*). Interestingly, in mock-transfected and Flag-tagged M1-transfected HEK293T cells, the COP1 protein displayed a broad distribution in the nucleus, with some speckles in the cytoplasm. When transfection with Flag-tagged N, the amount of COP1 in HEK293T cells increased markedly in cytoplasm, compared with mock-transfected and Flag-tagged M1-transfected cells (Fig. 4*C*). Collectively, these data indicated that PEDV N protein recruits the COP1.

### N protein amino acids 1 to 124 were required for COP1 recruitment and p53 degradation

**Figure 5:**
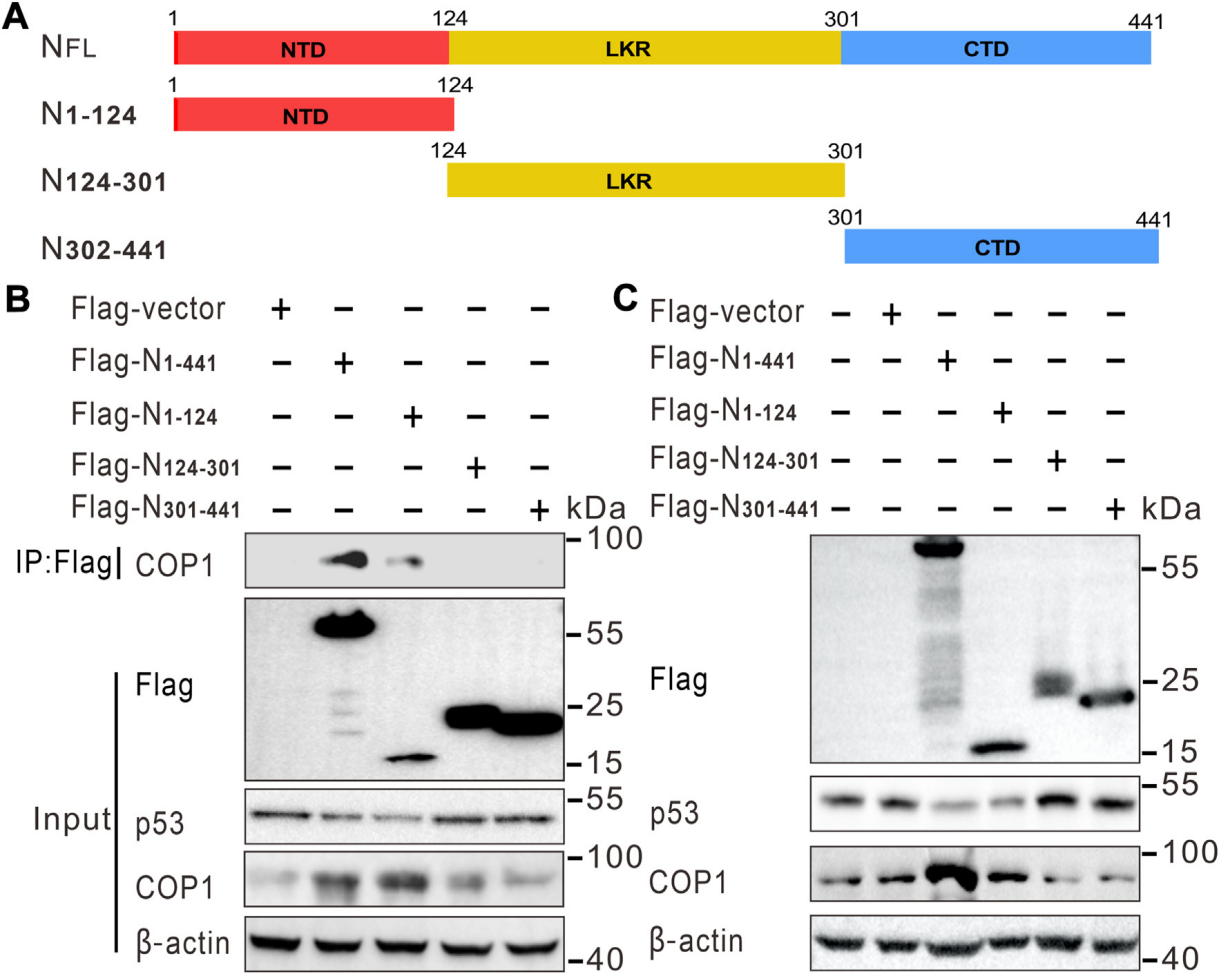
**N protein amino acids 1 to 124 are important for regulation of p53 levels and COP1 interaction.***A*, schematic domain structure of the full-length protein and serial deletion mutants of PEDV N used in the study. *B*, N-terminal domain of PEDV N (residues 1–124) were crucial for COP1 interaction. HEK293T cells were transfected with PEDV N proteins, encoding either full-length PEDV N (1–441) or different truncated versions (residues 1–124, 124–301, and 301–441), as indicated. All PEDV N proteins were tagged with Flag epitope. Cells were harvested at 24 h. 10% of the lysed cells were saved as input, and the remaining portions were subjected to immunoprecipitation with 1 μg anti-Flag antibody. The presence of COP1 protein in the immunoprecipitates was assessed by Western blotting. *C*, the full-length PEDV N or N-terminal domain of PEDV N promoted the p53 degradation and increased the protein level of COP1. Full-length Flag-N (residues 1–441), Flag-N (residues 1–124), Flag-N (residues 124–301), or Flag-N (residues 301–441) were expressed in HEK293T cells for 24 h. Subsequently, total lysates were immunoblotted to detect the expression of p53 and COP1. Flag-N, flag-tagged N protein; NTD, N-terminal domain; PEDV, porcine epidemic diarrhea virus.

Interaction of N with COP1 suggests the involvement of COP1 in N-mediated p53 ubiquitination and degradation. To map the domain of PEDV N protein that interacts with COP1, a series of Flag-tagged deletion mutants of N were constructed, including full-length N (residues 1–441), residues 1 to 124, residues 124 to 301, and residues 301 to 441 (Fig. 5*A*). All truncated mutants of N protein expression constructs were transfected into HEK293T cells. The immunoprecipitation results show that COP1 coimmunoprecipitated with full-length Flag-N as well as the NTD of N protein (residues 1–124, NTD) (Fig. 5*B*, left top panel, lanes 2 & 3), though the interaction between NTD and COP1 was weaker than that of full-length N protein. No coimmunoprecipitation was detected with the N middle region, residues 125 to 301 (Fig. 5*B*, left top panel, lane 4) or the N protein C-terminal region, residues 302 to 441 (Fig. 5*B*, left top panel, lane 5). These experiments suggested that N protein binds to the COP1 *via* its NTD. To further define the domain(s) of N protein important for p53 degradation and also to determine if the binding domain of N is essential for COP1 stability and p53 degradation, the effect of full-length N protein and three N truncated mutant expression on p53 stability were tested by Western blotting. Figure 5*C* reveals that overexpression of the full-length N or NTD could increase COP1 expression and p53 degradation (Fig. 5*C*, lane 3 *versus* lane 4). These findings clearly indicate that the NTD of N protein is necessary and sufficient for COP1 binding, ultimately leading to ubiquitination-mediated p53 degradation.

## Discussion

In this study, we explored the mechanism of PEDV-induced degradation of antiviral host factor p53. We clearly show that PEDV N protein promotes p53 ubiquitination and degradation through interaction with the cellular ubiquitin ligase COP1 *via* its NTD. Our study reveals a hitherto unrecognized link between N protein and p53 in PEDV infection, which may have therapeutic implications.

The p53 level changes dynamically during viral infection. Shen *et al.* (37) reported upregulation of p53 in influenza virus-infected Vero cells at 4 to 8 hpi and 14 to 18 hpi but downregulation at 10 to 12 hpi, and similar dynamics were also confirmed in Madin-Darby canine kidney cells. Hao *et al.* (27) showed that p53 was activated early in HEK293 cells during PEDV infection but degraded at later stages of infection. Similarly, in our study, we observed that PEDV infection reduced p53 expression in Vero E6 and IPEC-J2 cells at 24 hpi but enhanced its expression at 6 and 12 hpi (Fig. 1, *A*–*C*). It may be due to the different roles of viruses in differentially regulating host cells in the p53 signaling pathway. Our data suggest that p53 regulation is a complex process in PEDV-infected cells and that decrease of p53 level in PEDV-infected cells was mediated by proteasome pathway (Fig. 1, *D*–*F*), consistent with the findings reported elsewhere (27, 38). We moved further to dissect the process of p53 degradation in PEDV infection.

The continuous race between viruses and hosts has spawned different strategies to antagonize each other. Upon viral infection, the host elicits an antiviral state by producing IFNs or inducing apoptosis (39, 40, 41). Conversely, viruses have evolved to modulate the host response to benefit its replication. In coronaviruses, p53 is established as an intrinsic host restriction factor of PEDV (27), transmissible gastroenteritis virus (42), severe acute respiratory syndrome coronavirus (SARS-CoV) (43), SARS-CoV-2 (44), and human coronavirus NL63 (HCoV-NL63) (43). Accordingly, they have evolved specific means to hamper p53 functions by inducing its degradation. SARS-CoV applies papain-like protease (PLpro), a catalytic domain of the nsp3 to counteract p53. SARS-CoV PLpro interacts and stabilizes E3 ubiquitin ligase RCHY1 (also known as Pirh2), thereby enhancing ubiquitination and degradation of p53 (43). Similarly, HCoV-NL63 PLP1/2 also targets RCHY1 for p53 degradation (43). Additionally, another group found that the PLP2 of HCoV-NL63 induces the proteasomal degradation of p53 by interaction with the cellular ubiquitin ligase MDM2 (38, 45). For SARS-CoV-2, the main viral protease nsp5 repress the activity of p53 possibly by directly degrading p53 or p53 cofactors that are involved in the transcriptional activity of p53 (44). Poly-ubiquitination of p53 and 26S proteasomal degradation occur in the cytoplasm (46). One of the possibilities that the p53 negative regulators may function is to keep p53 in the cytoplasm for ubiquitination. PEDV N protein functions in this way by sequestrating p53 in the cytoplasmic compartment (Fig. 2*H*). 5-Fu was used to enhance the phenotype of p53 perinuclear accumulation by the N protein as it is a well-known activator of p53 nuclear translocation (33). These findings suggest that coronaviruses devote different portions of its large genome to promote p53 degradation. As far as we know, our data provide the first evidence for a key role of N protein in the regulation of p53 protein stability, upregulated briefly by N protein at 12 hpt, but decreased thereafter with the accumulation of N protein (Figs. 2*A* and 3*B*).

Many viral proteins modulate the ubiquitination status of substrates of choice by affecting the relevant E3 ubiquitin ligase stabilization (36). The viral IFN regulatory factor 4 of Kaposi's sarcoma-associated herpesvirus targets and stabilizes the MDM2 E3 ubiquitin ligase to facilitate the degradation of p53 (47). Porcine circovirus type 2 ORF3 protein interacts with porcine Pirh2 and inhibits its stabilization, thereby promoting p53 expression (48). SARS-CoV PLpro-mediated p53 degradation *via* stabilizing E3 ubiquitin ligase RCHY1 (43). Given that coronavirus N protein has not been reported to possess direct ubiquitinating or deubiquitinating activity, its interaction with the key E3 ubiquitin ligases involved in p53 ubiquitination were explored. Here, we show that the protein level of COP1, but not the other ligases tested, was dramatically increased in the presence of PEDV N protein (Figs. 3 and 4*C*). Such increase results from stabilization of COP1 because it was not transcriptionally activated by N protein expression (Fig. 3*C*). COP1 is a RING finger E3 enzyme, which can also mediate its own ubiquitination, targeting itself for proteasomal destruction (49). Our results show that the N protein stabilizes COP1 through inhibiting its ubiquitination (Fig. 3*D*). As a result, COP1-mediated ubiquitination of p53 was enhanced and p53 degradation stimulated. Moreover, knockdown of COP1 by siRNA significantly suppressed N-induced p53 degradation (Fig. 3*F*). This suggest that COP1 was involved in p53 ubiquitination mediated by N protein. Furthermore, we found that induced expression of N protein has no effect on the protein levels of endogenous MDM2, which is a critical p53 E3 ubiquitin ligase and has been proven to be involved in HCoV-NL63 PLP2-mediated p53 ubiquitination (38). However, enhanced p53 degradation should not be the only consequence of COP1 accumulation. Besides p53, the substrate targets of the E3 ubiquitin ligase COP1 include STAT3 (50), c-Jun (51), metastasis-associated protein 1 (52), and TORC2 (53). It can be hypothesized that during PEDV infection, the protein levels of various genes might also be downregulated.

Both PEDV N protein and COP1 could directly interact with p53 (19, 24). Here, we provide evidence of physical interaction between the N protein and endogenous COP1 in the HEK293T cells by reciprocal immunoprecipitation using anti-Flag or anti-COP1 antibody (Fig. 4). Furthermore, we found cytoplasmic colocalization of the N protein with COP1 by confocal microscopy. Thus, N can interact with both COP1 and p53 in the cytosol. It was reported that p53 binds the region at residues 171 to 194 of N protein (19), while COP1 binds its NTD region covering residues 1 to 124 (Fig. 5*C*). Combining our findings with published data, we speculate that the interaction between N protein and COP1 is independent of p53 binding. The N protein recruits both COP1 and p53 through distinct surfaces, thus triggering p53 ubiquitination and degradation.

Our work on reduced p53 in response to PEDV N protein expression seems to contradict to that by Su *et al.* (19) who showed that p53 abundance was increased in N protein expressing cells. There are several possibilities of such discrepancy. One possibility could be due to the experimental settings, *e.g.*, the amount of recombinant vectors transfected (affecting the protein expression level) and the time of sampling for analysis. In our time-course analyses in PEDV-infected or N-expressing cells, p53 did increase at the early time point (12 h after PEDV infection or N expression) but markedly reduced at 24 h after virus infection or from 24 to 48 h after transfection (Figs. 1, *A*–*C*, 2*A* and 3*B*). These results suggest that the N protein above a certain higher level is a negative regulator of p53. Another possibility could be the use of different tags: Flag of eight residues in our work and GFP of 238 residues in Su’s work. GFP-tagged Drp1 constitutes aberrantly stable, GTP-resistant supramolecular assemblies both *in vitro* and *in vivo* (54). EGFP fused to the N terminus of actin tends to form excessive actin filaments and affects both the cellular morphological and physiological phenotypes as compared to the C terminus–fused actin-EGFP (55). The CD36 proteins are expressed normally irrespective of the GFP tag at either the N or C termini. However, the two recombinant proteins showed a discrepancy in the uptake and surface binding of OxLDL (56). These findings caution against the presumption that GFP-tagging rarely interferes with native protein behavior and/or function (54). It is possible that N protein fused to GFP might be different from Flag-tagged N protein in its interaction with p53, thus affecting p53 stability.

In summary, findings presented here establish that PEDV N protein could induce the ubiquitin proteasome-dependent degradation of p53. This newly revealed function of N protein is mechanistically linked with its ability to promote p53 ubiquitination by recruiting and stabilizing COP1 through its NTD. Because p53 has been identified as an antiviral factor that constraints the replication of PEDV, its degradation caused by the interaction between N protein and COP1 may represent a viral strategy to contain the host constraining factor in favor of its propagation.

## Experimental procedures

### Cell lines and viruses

Vero E6 (African green monkey kidney cells, ATCC) or HEK293T (human embryonic kidney cells, ATCC) cells were grown in Dulbecco's modified Eagle's medium (Gibco) containing 10% fetal bovine serum (Gibco) under standard tissue-culture conditions (37 °C, 5% CO_2_). IPEC-J2 (porcine intestinal epithelial cells, ATCC) cells were cultured in Dulbecco's modified Eagle's medium–F12 supplemented with 10% fetal bovine serum under the same conditions. The PEDV classic strain CV777 (genotype Ⅰ [GⅠ], GeneBank accession number KT323979.1) and PEDV epidemic strain YJH-P141 (genotype Ⅱ [GⅡ], GeneBank accession number MT646162.1) were stored in our laboratory. They were propagated and titrated using Vero E6 cells.

### Reagents and antibodies

MG132 was obtained from Sigma-Aldrich and 5-Fu from Sangon Bio-Technology Co. All chemical compounds were dissolved in DMSO and stored at −20 °C. For some experiments, MG132, 5-Fu, or carrier DMSO were used at a concentration of 10 μM as detailed in the corresponding figure legends.

Mouse monoclonal anti-PEDV N protein was produced in our laboratory. The mouse monoclonal antibody against p53 (DO-1), COP1, and TOPORS were purchased from Santa Cruz Biotechnology and mouse anti-RCHY1, anti-Flag, anti-Myc, anti-β-actin, and rabbit anti-HA antibodies from Cell Signaling Technology. Rabbit monoclonal antibody against ubiquitin was acquired from Abcam. Horseradish peroxidase (HRP)-conjugated anti-rabbit IgG antibody and HRP-conjugated anti-mouse IgG antibody were purchased from Sigma-Aldrich.

### Plasmids, siRNAs, and transfection

The recombinant plasmids encoding full-length N protein and different truncated domains of N protein were subcloned into the p3×FLAG-CMV7.1 vector by homologous recombination using the ClonExpress Ultra One Step Cloning Kit according to the manufacturer's instructions (catalog no. C115-01; Vazyme). Vectors pCDNA3.1-Myc-p53, pCDNA3.1-HA-Ub, and pCMV7.1-Flag-M1 were kept in our laboratory. The siRNAs targeting COP1 were obtained from a referenced literature (24) and synthesized by GenePharma. Specifically, the COP1 siRNA sequences used were as follows: COP1 siRNA1 (AACUGACCAAGAUAACCUUGA), COP1 siRNA2 (AAGACUUGGAGCAGUGUUACU), and COP1 siRNA3 (AAGAGGUGUUGGAGUGUUGAC). Control siRNA in experiments was purchased from Genepharma. HEK293T were seeded into 6-well plates and cultured for 12 h. Cells were transfected with the above-described plasmids (2 μg per well) or siRNA using the jetPRIME transfection reagent (Polyplus) according to the manufacturer’s protocol and then incubated at 37 °C. Unless indicated, cells were harvested and lysed at 24 h after transfection.

### Virus infection

Twenty-four hours after seeding, Vero E6 cells were washed twice with PBS and then infected with CV777 or YJH-P141 at a multiplicity of infection of 0.01. After 1 h of PEDV adsorption, the cells were washed twice with PBS before adding 2 ml medium supplemented with 5 μg/ml trypsin with or without compounds as indicated until harvesting.

### Western blotting

Treated cells were washed with cold PBS and lysed in radioimmunoprecipitation assay (RIPA) buffer (Beyotime) containing a protease inhibitor cocktail (Bimake) for 20 min on ice, followed by centrifugation at 12,000 rpm for 10 min at 4 °C. Protein concentrations were measured using BCA Protein Assay Kit (Beyotime). The cell lysates were centrifuged at 12,000 rpm for 5 min at 4 °C and boiled for 10 min mixed with 5× SDS-PAGE sample buffer. Equivalent proteins were separated by SDS-PAGE and transferred to 0.45 μm polyvinylidene fluoride membranes (Merck Millipore) for Western blotting. The membranes were blocked with 5% nonfat milk in TBST buffer at room temperature (RT) for 1 h and further incubated with appropriate primary antibodies at RT for 2 h or overnight at 4 °C and then for 1 h with the appropriate HRP-conjugated secondary antibody. Proteins was visualized using enhanced chemiluminescence ECL (Bio-Rad) as described in the manufacturer’s instructions and analyzed with the Image J software. The protein levels were normalized by probing the same blots with β-actin antibody.

### Co-immunoprecipitation assay

HEK293T cells transfected with plasmids were rinsed with PBS (pH 7.4) three times and lysed in RIPA buffer (Beyotime) plus protease inhibitor cocktail (Bimake) on ice for 30 min. The cell lysates were centrifuged at 12,000*g* for 10 min at 4 °C to separate the RIPA soluble fraction from the pellet and incubated with the relevant primary antibody overnight at 4 °C with constant agitation which was followed by incubation of 4 to 6 h with Dynabeads protein G (Thermo Fisher Scientific). Then, the Dynabeads protein G were repeatedly washed with PBS. After that the bound proteins were eluted by boiling the beads in 30 ml of 2× SDS-PAGE sample buffer for 10 min and subjected to SDS-PAGE followed by Western blotting. The immunoprecipitates and whole cell lysates were analyzed by IB with the indicated antibodies.

### Immunofluorescence assay

HEK293T cells were cultured in 20 mm coverslips and transfected with the indicated expression plasmids for 20 h. Then, the cells were treated with 10 μM of 5-Fu or DMSO for 4 h. Following treatment, the cells were washed three times with PBS and fixed with 4% paraformaldehyde for 30 min at RT. Next, the fixed cells were permeabilized for 10 min with 0.5% Triton X-100 at RT and blocked for 30 min with 5% bovine serum albumin at 37 °C. After that, the cells were incubated with appropriate primary antibodies for 2 h at 37 °C or overnight at 4 °C. After the removal of unbound antibodies, cells were incubated with corresponding secondary Alexa Fluor antibodies (555 488; Invitrogen) for 30 min. To visualize the nuclei, DAPI (4′,6-diamidino-2-phenylindole) (Beyotime) was used to stain the cells at RT for 10 min. After washing the cells, fluorescence intensity was obtained using a confocal microscope (Olympus FV3000).

### Stability assay

Transient transfections were performed in HEK293T cells with plasmids expressing flag-tagged N or flag-tagged vector. At 24 hpt, cells were treated with 100 μg/ml CHX (Sigma-Aldrich) alone or with both CHX and MG132 for the indicated time periods, and lysates were analyzed by Western blotting.

### *In vivo* ubiquitination assay

For detection of ubiquitinated p53 protein, HEK293T cells (1.5 × 10^6^) were grown in 6-well plates and transfected with 2 μg of Flag-tagged N or Flag-M1 plasmids (as vector control plasmid). Twenty hours after transfection, cells were treated with MG132 (10 μM) for 4 h to block proteasome-mediated degradation. Then, cell lysates (supplemented with protease inhibitors) were prepared and subjected to immunoblotting with a monoclonal antibody against p53 (DO-1).

Equal amounts of various expressing plasmids HA-Ub, Flag-tagged N, and Myc-p53 (or Myc-COP1) were cotransfected into HEK293T for 20 h and treated for an additional 4 h with 10 μM of MG132 before harvesting. The cell lysates were prepared. The mixtures were immunoprecipitated by anti-Myc antibody. The purified Myc-p53 or Myc-COP1 were examined with anti-HA antibodies by Western blotting.

### Real-time quantitative PCR

For measurement of the COP1 mRNA level, plasmids were transfected into HEK293T cells for 24 h by using jetPRIME transfection reagent (Polyplus). Total RNA was extracted from cells after different treatments by using TRIzol reagent according to the instructions of the manufacturer (Thermo Fisher Scientific). One microgram of RNA was reverse transcribed to cDNA using the HiScript III All-in-one RT SuperMix Perfect for qPCR (Vazyme), and the synthesized cDNA was analyzed by RT-qPCR using Taq Pro Universal SYBR qPCR Master Mix (Vazyme) according to manufacturer’s the protocol. Primers used for RT-qPCR are COP1 primers forward ACCATTTGGCTTTCGG and reverse GCTTTACGGTGTCCT; GAPDH primers, forward TCACTGCCACCCAGAAGACTG and reverse GGATGACCTTGCCCACAGC. GAPDH gene was used as an internal control. Three independent experiments were performed for statistical analysis. The relative fold change was calculated using the 2^−ΔΔCt^ method after normalizing with GAPDH.

### Statistical analysis

Data were presented as mean ± standard deviation. Statistical evaluation was conducted by Student’s *t* test using GraphPad Prism 6 software. A probability (*p*) value of <0.05 was considered statistically significant. All experiments were performed in triplicates.

## Data availability

All data are contained within the article.

## Conflict of interest

The authors declare no conflict of interest with the contents of this article.
